# A paramedic’s role in reducing number of falls and fall-related emergency service use by over 65s: a systematic review

**DOI:** 10.29045/14784726.2021.6.6.1.46

**Published:** 2021-05-01

**Authors:** Mhairi Bonner, Matt Capsey, Jo Batey

**Affiliations:** West Midlands Ambulance Service University NHS Foundation Trust; Teesside University ORCID iD: https://orcid.org/0000-0003-3659-5344; South Tees Hospitals NHS Foundation Trust

**Keywords:** accidental falls, ambulance, referral

## Abstract

**Background:**

Around 10–25% of emergency calls for adults aged over 65 are attributed to falls. Regardless of whether injuries are caused, quality of life is often affected by fear of falling, leading to reduced confidence and activity, negatively impacting mobility and risking depression and isolation. Ambulance service staff are well placed to identify falls risk factors so patients can be directed to falls prevention services. This article aims to determine how the referral by paramedics of uninjured falls patients to community falls services may reduce future falls and emergency services use.

**Methods:**

The CINAHL, MEDLINE and AMED electronic databases, grey literature sources and reference lists of relevant papers were systematically searched to identify primary research of an experimental design. Studies were eligible if they included elderly patients, aged over 65, who had received a paramedic response following a fall, were found to be uninjured and who were referred to local falls services rather than being transported to hospital. The study outcomes were required to include the rate of subsequent falls and emergency service use.

**Results:**

Four papers from three studies were included in the review following quality assessment. Results were not always statistically significant but showed a reduction in subsequent falls, particularly in the high-risk population, and in emergency service call-outs. A consistent positive effect was seen on the patients’ well-being and independence related to activities of daily living across the studies.

**Conclusions:**

The relationship between paramedics and local falls services has changed the pre-hospital management of these patients. Generally, access to a falls-specific care package has proved beneficial in supporting independence and reducing unnecessary transport to hospital. Further research into the uptake of this care pathway by paramedics now it is more established may be useful, as would research into barriers to adherence of the elderly to such an intervention.

## Background

Public Health England (2018) defines a fall as an event causing a person to land on a lower level unintentionally without the occurrence of a major intrinsic event such as a stroke. Around a third of people aged over 65, and half of those over 80, fall at least once a year (Public Health England, 2018). It is expected that around 20 million people will be aged over 60 by 2031 (NHS Confederation, 2012) compared to around 15.3 million in 2018 (Age UK, 2019). Falls account for 10–25% of emergency ambulance responses for over 65s each year (NHS Confederation, 2012), and so the demand on ambulance services is likely to increase. Regardless of whether injuries result, fear of falling often affects quality of life in the elderly, leading to reduced confidence and activity, negatively impacting mobility and risking depression and isolation (Taguchi et al., 2015).

Risk factors for falls in over 65s include frailty, conditions affecting mobility or balance such as diabetes or arthritis and cognitive or visual impairment (NICE, 2019). Fear of falling and a fall within the last 12 months are also included, as are medications, particularly the use of benzodiazepines, anti-hypertensives or where more than four medications are used regularly regardless of the type. The work of paramedics in the community puts them in a good position to identify people at risk and direct them into services that aim to reduce falls such as through direct referral to a community-based falls service when a patient is uninjured following a fall.

Some ambulance services across the UK have implemented an alternative approach where a vehicle manned by a paramedic and an occupational therapist responds only to falls. This facilitates a timely response and patient assessment, followed by an immediate environmental assessment by the occupational therapist if conveyance to the emergency department is not appropriate. This aims to speed up implementing measures that enable more patients to remain at home safely, reducing the likelihood of future falls and fall-related hospital admissions (Bedwell & Tracey, 2016). In addition to its role in improving the quality of care from the patient’s perspective, financial benefits of this initiative have also been identified.

The efficiency of falls management involving paramedics has been explored from a number of angles. For example, the financial implications of establishing a new care pathway compared to the cost of the traditional route, involving conveyance of all patients to the local emergency department, have been investigated to identify the long-term sustainability of the option (Snooks et al., 2014). Qualitative research can also be found and shows positive responses when exploring the attitudes and opinions of both patients and paramedics on how this option is working (Pyer et al., 2015). Systematic reviews have been published on this topic; however, a gap in the research exists when looking at the effect on subsequent falls and emergency service use from a quantitative perspective as the management of these patients has developed since publication of the latest relevant review (Zozula et al., 2016).

This article aims to answer the question: How does paramedic referral of elderly patients, aged over 65, and uninjured following a fall, to a community falls team affect the rate of future falls and emergency services use by these patients?

## Methods

The CINAHL, MEDLINE and AMED electronic databases were systematically searched using the research question on 5 October 2020 and 13 November 2020.

When determining key words to use for the search, general terms for people who fall and the elderly were used, aiming to return a broad set of results that could be narrowed down by more specific terms for the intervention regarding ambulance staff referral and falls care pathways. Boolean operators were used to combine the search terms as seen in Supplementary 1, and limiters for a date range and type of research were considered but not used due to the manageable number of results returned and the relatively recent introduction of this type of care pathway.

The database search results were added to those from grey literature searches, applying the same question to Google Scholar, OpenGrey and NICE Evidence, also on 5 October 2020. Finally, hand searching of the reference lists of relevant papers was completed following each search.

Where abstracts were found as search results but did not include the full research paper, authors were contacted by email and phone in an attempt to obtain the full paper for consideration.

Where the title of a search result appeared relevant to the research question, the abstract was read to determine whether each element of the research question was addressed.

Inclusion criteria included the population of interest, people aged over 65 who received an emergency ambulance response and were uninjured following a fall. The intervention was a direct referral by a paramedic to a community-based falls service providing a multidisciplinary assessment and management programme. Elements of these include strength and balance training, home risk assessment and a medication review (NICE, 2017).

Comparisons included traditional ambulance service care, such as conveyance to the local emergency department, unless the patient refused, or discharge on scene without follow-up care in place. Studies where follow-up was arranged with other healthcare professionals, such as the patient’s GP or regular carers, were also permitted as these do not involve the multi-factorial approach to management that comes with referral to a falls service. Outcomes were required to include the rate of subsequent falls and emergency service use by participants. The objective of this review requires quantitative data for analysis, so qualitative papers were excluded.

Paramedics within UK and American ambulance services both have the option to discharge patients on scene and have similar rates of non-conveyance of fall patients (Snooks et al., 2006). Research has also been systematically reviewed from healthcare services in Australia, as falls patients comprise a significant proportion of the workload of ambulance services in New South Wales, where there is also the option of non-conveyance in the absence of injury or other abnormal clinical findings (Mikolaizak et al., 2013). Therefore, UK, American and Australian research papers that fit the inclusion criteria are included in this review.

If these criteria were met, the full paper was obtained, and its quality assessed using the Critical Appraisal Skills Programme (2018) checklist appropriate to the type of study.

## Search results

**Figure 1:**
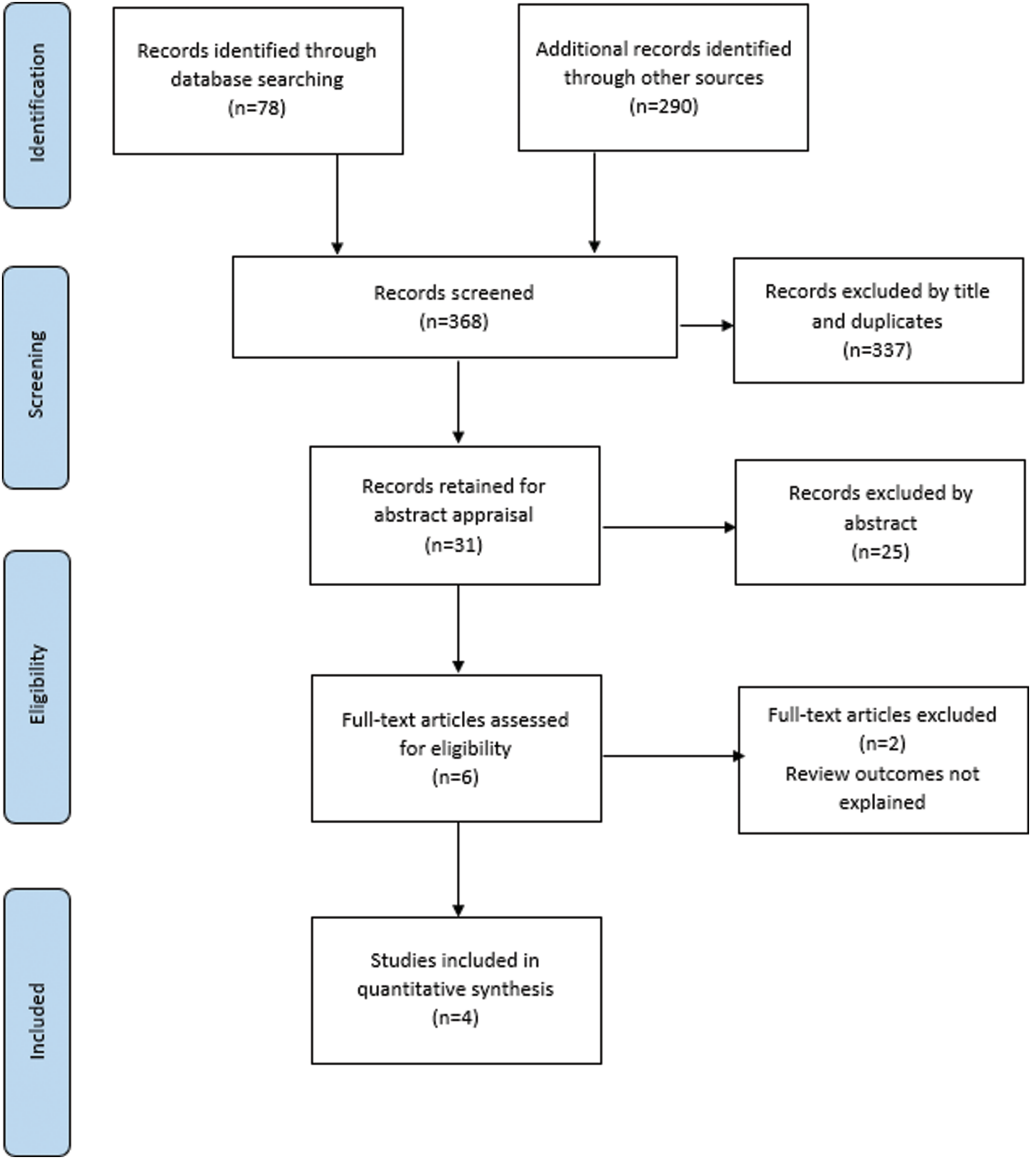
Figure 1. PRISMA flow diagram, based on Moher et al. (2009).

The search identified 368 papers; 337 were excluded as duplicates or due to irrelevant titles. The abstracts of the remaining 31 papers were reviewed and 25 were discarded, as were two further papers as the outcomes relevant to this review were not fully explained (Figure 1). The search highlighted a recently developed option involving a combined occupational therapist and paramedic response vehicle through two abstracts from the College of Occupational Therapists Conference in 2016 and 2017 (Bedwell & Tracey, 2016; Carlill & Puddy, 2017) that were relevant to the research question. However, as it was unclear whether full papers existed for these results and attempts to contact the authors were unsuccessful, they had to be excluded from the review and this option could not be explored further at this point.

The final four papers were all randomised controlled trials. Following quality assessment, these were included in the review and are summarised in Table 1.

**Table 1.** Details of included studies.

Author(s)	Title	Year of publication	Number of participants
Logan et al.	Community falls prevention for people who call and emergency ambulance after a fall: randomised controlled trial	2010	204
Mikolaizak et al.	A multidisciplinary intervention to prevent subsequent falls and health service use following fall-related paramedic care: randomised controlled trial	2017	221
Snooks et al.	Paramedic assessment of older adults after falls, including community care referral pathway: cluster randomised trial	2017	4655
Snooks et al.	Support and assessment for fall emergency referrals (SAFER)2: a cluster randomised trial and systematic review of clinical effectiveness and cost-effectiveness of new protocols for emergency ambulance paramedics to assess older people following a fall with referral to community-based care when appropriate	2017	

Logan et al. (2010) was a UK-based study of adults aged over 60 who were not transported to hospital following ambulance attendance for a fall. The intervention was a multi-factorial intervention plan based on UK clinical falls guidelines in place at the time (NICE, 2004), primarily delivered in the home with optional group sessions. A comparison group was included, receiving no further intervention after recruitment. Logan et al. (2010) confirmed their hypothesis that the intervention would reduce the rate of falls over 12 months when compared to usual practice (see Table 2). The results returned a larger effect than they anticipated but the population was believed to be less functionally able at baseline than in other similar studies.

**Table 2.** Results of included studies.

Study	Follow-up period	Outcome	Intervention group	Control group	Effect size IRR (95% CI)	P value
Logan et al. (2010)	12 months	Falls per person per year/no. participants	3.46/102	7.68/102	0.45 (0.35–0.58)	< 0.001
		Ambulance call-outs (fall related)	245	365	0.60 (0.40–0.92)	0.018
Mikolaizak et al. (2017)	12 months	Falls/no. participants	306/111	271/110	1.18 (0.86–1.61)	0.320
		Ambulance call-outs (fall related)	136	108	1.27 (0.87–1.84)	0.214
					**Odds ratio (95% CI)**	
Snooks et al. (2017a, 2017b)	1 month	Falls/no. participants	413/621	409/589	0.723 (0.544–0.961)	0.025
		Ambulance call-outs (reason unknown)	442	493	0.815 (0.705–0.943)	0.006
	6 months	Falls/no. participants	228/329	192/296	1.495 (1.014–2.205)	
		Ambulance call-outs (reason unknown)	1046	1046	0.899 (0.799–1.011)	0.076

CI = confidence interval; IRR = incidence rate ratio.

Mikolaizak et al. (2017) studied adults aged over 65 who received a fall-related emergency response. The intervention was a multi-factorial response using existing services to address the individual’s risk factors for falls following assessment organised by the researcher. A comparison group was included, receiving written advice from the researcher on how to address their risk factors and advised to speak to their normal healthcare provider for further assistance.

All included studies performed intention to treat (ITT) analysis (see Table 2). Mikolaizak et al. (2017) reported that their multi-factorial intervention did not prevent falls in the population. However, it suggests adherence is a barrier to effectiveness. This was explored through per-protocol analyses (Table 3) where ‘adherers’ were defined as participants that completed all recommended interventions, of which there were 39 in the intervention group (46%). When comparing the ‘adherers’ to ‘non-adherers’ in the intervention group, a significant difference was found in the subsequent number of falls and fall-related ambulance call-outs. If high levels of adherence to recommendations were achieved, it could still be an effective strategy to reduce falls and subsequent healthcare service use. Although a greater difference in the outcomes was seen in the subset of ‘adherers’ when compared to the whole control group undertaking written advice, these were not statistically significant.

**Table 3.** Mikolaizak et al. (2017) per-protocol analysis.

Per-protocol analysis			Unadjusted analysis		Adjusted analysis	
	‘Adherers’ (n = 39)	‘Non-adherers’ (n = 46)	IRR (95% CI)	P value	IRR (95% CI)	P value
Falls	87 (2.06)	189 (3.94)	0.53 (0.32–0.86)	0.011	0.53 (0.32–0.87)	0.012
Ambulance call-outs (falls related)	39 (0.92)	87 (1.82)	0.51 (0.29–0.91)	0.022	0.53 (0.30–0.94)	0.030
	**‘Adherers’** (n = 39)	**Control group** (n = 110)				
Falls	87 (2.06)	271 (2.72)	0.76 (0.49–1.20)	0.240	0.75 (0.47–1.17)	0.203
Ambulance call-outs (falls related)	39 (0.92)	108 (1.16)	0.88 (0.52–1.50)	0.638	0.84 (0.49–1.43)	0.512

CI = confidence interval; IRR = incidence rate ratio.

The final two papers reported on the same UK clinical trial. They were grouped together for the purpose of results discussion. The trial involved participants aged over 65 years who received a fall-related emergency response from paramedics (Snooks et al., 2017a, 2017b). The intervention involved a clinical protocol for paramedics to refer patients who had fallen to a community-based falls service when transport to hospital was not required. The falls service enabled them to receive the multi-factorial intervention plan. A control group was used for comparison of normal paramedic management, including transport to hospital unless the patient refused. Snooks et al. (2017a, 2017b) concluded that there were no differences between the trial groups in the use of healthcare services other than a small reduction in subsequent emergency calls (Table 2). Referral rates were lower than expected, which may have contributed to this. However, it was shown that a new referral option could be introduced safely for this type of patient, and that paramedics in the intervention group left fewer patients at home without ongoing care in place, which may have contributed to the lower number of subsequent emergency calls in the intervention group.

### Outcome/comparison of results

ITT analysis by Mikolaizak et al. (2017) showed no significant reduction in subsequent falls or healthcare service use over the follow-up period, although a reduction was seen in per-protocol analysis. Mikolaizak et al. (2017) suggest that the multi-factorial intervention may have been overwhelming to participants, reducing adherence and its overall impact. The activity levels of ‘adherers’ may also have increased, potentially resulting in fatigue and greater exposure to falls risk situations. This, in addition to the high adherence rate to written recommendations in the control group, may have contributed to the lack of between-group differences (Mikolaizak et al., 2017). No differences in quality of life between the groups were discovered during the study period.

Conversely, Logan et al. (2010) reported a 55% reduction in subsequent falls in their intervention group. This was larger than expected and may be attributed to the high-risk sample, reporting an average of two falls in the previous three months and less functional ability than previous similar studies based on Barthel Index scores. To ensure this was a genuine treatment effect, the researchers closely examined the treatment given and found that over 79% of intervention participants received at least seven intervention sessions, in line with falls clinical guidelines (NICE, 2004). Given the significant reduction in falls, it is not surprising that significantly fewer fall-related emergency service calls were also reported. Improvements were also seen in activities of daily living of the intervention group through higher Barthel Index and Nottingham Extended Activities of Daily Living scores, as well as a reduction in fear of falling shown by the falls efficacy scale. This contributes to the positive effects of this approach to falls prevention, although as the intervention is a package of care, it cannot be determined if individual aspects were particularly effective and others less so.

Snooks et al. (2017a, 2017b) found little difference between trial groups in their overall composite outcome of subsequent emergency episodes (including emergency service calls, emergency department attendances, emergency admissions and death) at the one- or six-month point, but significantly fewer emergency calls were made by the intervention group at one month (p = 0.006) and non-significantly fewer at six months (p = 0.076). Significantly fewer falls were reported after one month by the intervention group (p = 0.025), but after six months this group reported more falls than the control group. This could suggest that the intervention was helpful in the short term but the effect did not last. Combined with the results for subsequent emergency calls, it could be suggested that although the participants still suffered falls, they were perhaps better prepared to cope with these supported by their falls teams rather than require an emergency response. However, this study does not differentiate between subsequent emergency calls due to falls and other reasons, so the true effect of the intervention on these is unclear. Intervention participants also reported higher satisfaction with the interpersonal aspects of care. Fewer were left at the scene without ongoing care compared to control group participants, despite the low referral rate to falls services of only 8%. This shows the positive effect of this treatment plan on the well-being of patients, which may also contribute to their confidence and quality of life, reducing their falls risk.

### Limitations

Originally used as an academic piece of work, this review was required to be completed by an individual researcher as a university dissertation. Therefore, it is vulnerable to researcher bias as a team of researchers could not be consulted when choosing studies for inclusion or reporting results. To minimise this risk, an academic supervisor was assigned by the university and used by the researcher to gain a different viewpoint and advice when planning the search strategy and interpreting results of studies where required.

Also, neither the individual researcher nor academic supervisor had detailed knowledge of the role of occupational therapists as the main profession delivering the intervention. Therefore, input from a local occupational therapist was permitted to provide access to specialist background knowledge and resources.

As a small-scale research project, a further limitation of this review was the availability of resources. As this was an academic piece of work, university access was provided to enable the database searches and further support was provided by the academic supervisor in updating this review following its university submission.

One final limitation of this review was the identification of a joint occupational therapist and paramedic response vehicle as a management option for these patients. This is a fairly new initiative used in a limited number of areas and, due to this, only two conference abstracts were identified by the search which had to be excluded due to the lack of a full research paper. This resulted in a treatment option that met the reviews aim being entirely excluded.

### Implications for practice

The multi-factorial approach to falls prevention is recommended by NICE (2013) to address common falls risk factors, and this review has shown the intervention can be effective when the treatment plan is adhered to, particularly in those at high risk. Results for subsequent falls and emergency service use due to falls varied, but a consistent positive effect on patients’ well-being was found. This can be considered to contribute to reducing further falls by improving individuals’ confidence and therefore engagement in activity and treatment. In the future, further research into adherence by older people to a similar intervention may be beneficial, as suggested by Mikolaizak et al. (2017), as a greater understanding of this may facilitate alterations to the delivery of falls prevention interventions, encouraging greater adherence and therefore, greater benefit. Findings from this type of research may also be used to improve adherence to other aspects of care in a similar way. Following Snooks et al. (2017a, 2017b) finding low referral rates, it may be beneficial to repeat a study on the uptake of this care pathway by paramedics now it is more established and widely used, including the identification of any barriers that limit its use.

## Conclusions

This systematic review identified a significant amount of falls prevention research for older adults, which when narrowed down to those involving a paramedic assessment, showed the changes in practice relating to the management of these patients. Ultimately, four papers were included relating to the relationship between paramedics and local dedicated falls services. Generally, fewer falls and subsequent emergency calls were made by the intervention groups, although the difference was not always statistically significant. This, along with the improvements to well-being and activities of daily living noted by the included studies, supports the finding that access to a falls-specific care package is beneficial for older people who fall in supporting their independence and reducing unnecessary transport to hospital, but there are still areas that would benefit from further research.

## Author contributions

The review was undertaken and reported by MB with contributions by MC for research methods, results interpretation and structure of the report. JB advised on current occupational therapy practices in her region and occupational therapy resources that were searched as grey literature, and assisted in attempts to contact specific authors. MB acts as the guarantor for this article.

## Conflict of interest

None declared.

## Ethics

Not required.

## Funding

None.
